# Latent Diseases

**Published:** 1909-04-03

**Authors:** 


### LATENT DISEASES.

Extracts from a Lecture by the late PROFESSOR POTAIN, M.D., of Paris.

(*Specially reported for* The Hospital.)

By latent diseases, we mean those diseases the existence of which is suspected neither by the patient nor by the physician, because their development being exceedingly insidious or because they assume the aspect of some quite different affection.

Recently a woman was admitted into the wards in a state of complete asystolism. She had been treated in 1894 for rheumatic endocarditis, and at that time I had noted the following symptoms: At first an obscuration, and finally the complete disappearance of the second sound and the appearance of a systolic souffle. The nature and the seat of these signs showed the existence of inflammation of the aortic and mitral valves. The lesions of the aortic orifice, however, were only transitory, and gradually the sounds became quite normal again; but the mitral souffle persisted, although much slighter than at first. The patient then left the hospital.

On the present occasion she showed all the signs of considerable cardiac hypertrophy, which was rather surprising since the mitral souffle was so slight that there could not be a lesion sufficient to account for the marked degree of hypertrophy; and, moreover, the latter generally arises at a very late period in mitral lesions, contrary to what happens in aortic trouble. The autopsy gave us the key to this unusual state of things, for we found extreme adherence of the pericardium. The adhesions were formed by thick fibrous tissue, which at certain points had become almost cartilaginous. The mitral valve was very irregular and presented numerous milky patches. The water test showed regurgitation, and there was at the same time a certain degree of stenosis owing to adhesion of the edges of the valves.

We thus see that in this patient the principal lesion, the one which had determined the cardiac hypertrophy, consisted in the pericardial adhesions, and these had developed in an absolutely quiescent way. It is very important for practitioners to know that latent diseases exist, and to learn, as far as it is possible to do so, how to detect them.

The first thing that strikes one when investigating this subject is the great importance of semiology. It is not sufficient to know that such and such an affection gives rise to such and such symptoms, but it is necessary to differentiate carefully which of these symptoms are common to other diseases.

Symptoms may be naturally divided into two classes; first, functional, and second, physical. Speaking generally, the functional symptoms are those which first attract the notice of the patient, and for which he comes to consult us. The physical symptoms are those which the physician alone is competent to detect and recognise, and he only looks for them when the nature of the functional symptoms indicate the probability of some morbid process existing.

Of course, it is not always possible to detect physical signs when dealing with diseases of deep seated and inaccessible organs, such as the brain and spinal cord, for example, and in these cases functional symptoms are of prime importance. The latter, however, may themselves be absent, and it is easily understood how certain affections may run their course, even to the very end, in an insidious and quiescent manner, without giving rise to any symptoms, subjective or objective.

Latent diseases may be divided into three classes: (1) Latent diseases proper, namely, those which give rise to no symptoms whatever; (2) masked diseases; (3) dissimulated diseases.

In the first class we have certain cases of cerebral tumours, of cancer of the kidney, of pericardial adhesions, of gastric ulcer, of renal calculi. In the Museum of the Faculty of Montpellier there is preserved a man's brain with a knife blade embedded in it, without there having been any cerebral symptoms before the man's death. It is not at all rare to find, at the autopsy of elderly people, calculi in the kidneys which had not given rise to any symptoms during life. I remember a case of large encysted calculus of the kidney filling the whole of the pelvis of the organ without there having been the slightest symptom of its presence. These cases are explained by the greatly diminished sensibility of the tissues in elderly people. Generalised miliary tuberculosis of the lung may run its whole course without betraying its existence. Adherent pericardium or adherent pleura may, for a very long time, remain latent, and this is also true in certain cases of chronic atrophic nephritis.

Many affections, the anatomical substratum of which consists in atrophy or stricture of an organ, only become apparent when the patient puts more work than usual on that particular organ. I have called this state of diminished functional power "miopragia" (Gr. μεῖον diminution). Gibert, of Havre, has noted that children suffering from stenosis of the bowel keep well as long as they take only milk, but as soon as solid food is given trouble arises. Mitral stenosis gives rise to a state of circulatory miopragia, which only becomes manifest when the patient, doing more work than usual, increases the flow of blood through the heart.

As an example of "masked diseases" I might give chronic diarrhœa of malarial origin. I have come across quite a number of cases of most persistent diarrhœa which only disappeared on the administration of quinine. Malaria may assume other masks. A man suddenly becomes comatose, and the case is diagnosed as apoplexy; as, however, there is a past history of malaria, quinine is injected subcutaneously, and the man recovers rapidly.

The marked preponderance of a single symptom frequently gives rise to error in diagnosis. I have often had occasion to quote the following case: One of my surgical colleagues was for a long time considered to be suffering from some serious cerebral trouble; he was subject to attacks of vertigo of such intensity that, when going round the wards, he often had to clutch hold of the bedrails to save himself from falling. Careful auscultation showed that it was a case of aortic regurgitation, and that the symptom "vertigo" had overshadowed all others.

The third class of latent diseases, *i.e.* simulated and dissimulated affections, is the largest of the three, and contains a great number of very different affections. I shall not do more than mention that class of diseases which are simulated or hidden by patients either for no apparent reason (hysteria) or for some advantage to be gained (malingering). Much more interesting are the diseases which are masked by the co-existence of some other affection, or by the presence of some complication. Pulmonary emphysema, which frequently accompanies phthisis, may mask the physical signs of the latter. The diagnosis of aortic regurgitation becomes very difficult when there is at the same time mitral stenosis. The existence of gastric affections is often ignored by patients. A medical man of my acquaintance who suffered from cardiac symptoms got entirely rid of these by a simple lavage of the stomach. It was the latter which gave rise to the heart symptoms, and yet digestion seemed to take place quite normally and the patient had not noted any gastric symptoms whatever.

It is not at all exceptional for a gastric affection to give rise to all the symptoms of heart disease: palpitations, dilatation of the right ventricle and auricle, congested liver, ascites, œdema, etc., and all these may disappear when appropriate treatment is given for the stomach trouble. If diseases of the stomach can influence the organs of circulation, on the other hand cardiac affections may give rise to gastric trouble, and the latter may be so marked as to overshadow all other symptoms. I have come across cases of persistent diarrhœa which have resisted all ordinary treatment, and auscultation has shown the presence of some circulatory affection. I saw a case of this sort with Dr. Guyot; the patient complained only of chronic diarrhœa. On examination I found an aneurysm of the abdominal aorta. Cardiac affections may simulate disease of the liver when congestion of this organ, ascites and œdema, predominate in the clinical tableau; they may also, particularly mitral lesions, cause marked nervous symptoms leading to errors of diagnosis.

In affections of the stomach one may observe vertigo, violent cephalalgia, and sometimes apoplectiform attacks. I remember the case of a patient presenting the latter symptom which was simply due to the absence of teeth and imperfect mastication of food. Gastric cough, if it happens to coincide with a certain degree of emaciation, may cause suspicion of early phthisis. Certain pseudo-asthmatic attacks, due either to bronchial spasm or vascular spasm or even to urticaria of the bronchial tubes, have a gastric origin.

Intestinal parasites may give rise to a variety of symptoms. In the adult, tænia may produce vesaniæ, kleptomania, etc. As soon as the parasite is expelled the mental symptoms disappear. As an example I might quote the following case. A naval officer, noted for his reckless daring and courage, became all of a sudden timorous, irresolute, and incapable of giving the most trivial order without experiencing a feeling of extreme nervousness. The ships' doctor, very much astonished at this marked change in the man's character, examined him carefully and found that he had a tapeworm. The latter was easily got rid of, and immediately the officer became his former self again. Intestinal affections may have a repercussion on the heart. I have seen entero-colitis give rise to symptoms of heart disease. Hepatic affections may have a more or less marked influence on the nervous system; hypochondria, for instance, may be produced. They may also cause heart or lung symptoms. I have seen a case of hydatid cyst of the postero-superior part of the liver in a young man give rise to a dry cough and progressive emaciation; incipient phthisis was feared.

Gall-stones may simulate cancer of the pylorus. A patient was considered to be suffering from cancer of the stomach and an operation had been arranged for. Having seen her, I made the diagnosis of cholecystitis due to calculi. The patient died before operation, and the post-mortem showed the presence of a calculus which had determined inflammation of the gall-bladder and peritonitis. I saw another case with Gueneau de Mussy. We had found a tumour in a patient with a cancerous heredity, and, as a matter of fact, she actually did die of cancer, but at a much later period. At the time we examined her she had no cancer, and the proof is that, having sent her to a Spa, she returned quite well and brought a whole bottleful of gall-stones which she had passed. Different lesions in a given organ may cause identical symptoms, and the diagnosis is often a very delicate matter. Nothing is more difficult to distinguish than anæmia and congestion of the brain, and Audral and Gasset describe the symptoms as identical. The mechanism differs, but the result is the same; in the first case there is not enough blood in the vessels and in the second there is too much.

Cerebral hæmorrhage and softening of the brain can only be distinguished when the circumstances are taken into account. If the patient has been suffering from chronic nephritis, one is more inclined to favour the diagnosis of hæmorrhage; if his arteries are atheromatous, softening is more probable. In the lungs it is often difficult to distinguish cancer from tubercle.

Confusion in the diagnosis of lesions in quite different organs may also exist, when they give rise to similar symptoms. Congestion of the lung may produce syncope and apoplexy, just as congestion of the brain does. It frequently happens for elderly persons to be struck down with apoplexy, and at the autopsy to find pneumonic consolidation. The same thing may happen when large pulmonary embolisms suddenly obstruct circulation.

Incipient phthisis sometimes gives rise to palpitations which may cause one to suspect heart disease. At a more advanced stage the same error may be caused by a certain degree of induration of the lung, producing increased præcordial dulness, which one takes to be due to hypertrophy of the heart. Difficulty of breathing and œdema of the lower extremities, due to lung affections, may also be referred to the heart. Dulness, due to sclerosis of the apex of the lung, may cause one to think of an aortic lesion.

If I seem to have developed my subject more than is necessary, and if I have given many cases as examples, it is only to show how necessary it is to examine patients most carefully and minutely.

In conclusion I may say that if functional symptoms often cause errors of diagnosis, physical signs, although of greater importance and value, are themselves subject to caution and require to be carefully studied before drawing any inferences from them.

